# Comparison of Retention Behavior between Supercritical Fluid Chromatography and Normal-Phase High-Performance Liquid Chromatography with Various Stationary Phases

**DOI:** 10.3390/molecules24132425

**Published:** 2019-07-02

**Authors:** Tsunehisa Hirose, Daniel Keck, Yoshihiro Izumi, Takeshi Bamba

**Affiliations:** 1 Nacalai Tesque, Inc., Ishibashi 617-0004 17, Kaide-cho, Muko-shi, Kyoto, Japan; 2 Division of Metabolomics, Medical Institute of Bioregulation, Kyushu University, Maidashi 812-8582 3-1-1, Higashi-ku, Fukuoka, Japan

**Keywords:** supercritical fluid chromatography, normal-phase high performance liquid chromatography, stationary phase, retention behavior, dispersion interaction

## Abstract

The retention behavior of a wide variety of stationary phases was compared in supercritical fluid chromatography (SFC) and normal-phase high-performance liquid chromatography (NP-HPLC). We also attempted to elucidate the retention behavior in SFC by investigating the selectivity of the different stationary phases. SFC separation conditions with polar stationary phases, such as silica gel (SL) and diol (Diol) phases, operate via adsorptions that include hydrophilic and ionic interactions similar to those in NP-HPLC. Moreover, non-polar stationary phases, such as pentabromophenyl (PBr), pyrenylethyl (PYE), and octadecyl (C_18_), could be used despite the non-polar mobile phase conditions, because the dispersion and π-π interactions were stronger in SFC than in HPLC. These results reflect the selectivity of the stationary phase and its retention factor, thus providing useful information for the selection of appropriate stationary phases for particular analytes.

## 1. Introduction

Similar to high-performance liquid chromatography (HPLC), supercritical fluid chromatography (SFC) is not a novel technology. However, recently, as major HPLC instrument manufacturers have entered the SFC market and innovative instruments have been developed, the number of SFC users has rapidly increased. Owing to high-speed and preparative separations associated with SFC, the change from HPLC to SFC is promising for both chiral and achiral separations. It was recently demonstrated that almost the same degree of robustness is possible in SFC as compared to HPLC by proper adjustments of operational conditions [1]. However, the number of HPLC users is higher than SFC users, especially for achiral separations. SFC is positioned as an alternative method in cases where HPLC yields insufficient separation and as a complementary process to HPLC.

As an alternative or complementary method, the selectivity of SFC must be different from that of HPLC to improve separation. Thus, if the same selectivity in the SFC and HPLC procedures is used, the overall utility of the method is reduced. It is possible that, because SFC uses supercritical fluids as the mobile phase, selectivity will differ from that of HPLC. The selectivity of SFC can be controlled by changing the mobile phase composition, temperature, and pressure [2,3,4,5,6], but the stationary phase most significantly affects selectivity. In addition to the number of stationary phases specially designed for SFC, stationary phases used in HPLC procedures are also used for SFC, making it is difficult to select the optimal stationary phase. In SFC, as no stationary phase has demonstrated overwhelming versatility like the octadecyl (C_18_) phase of reversed-phase high-performance liquid chromatography (RP-HPLC), a typical user must select the stationary phase on a trial and error basis. Chromatographic analyses of drugs, foods, metabolites, and polymers have been discussed in several reviews [7,8,9,10,11,12,13,14,15], but the stationary phases used are often disjointed, even for the same sample. For example, C_18_, phenyl (Ph), cyanopropyl (CN), diol (Diol), ethylpyridine (EP), and silica-based stationary phases are all commonly used.

As an effective column selection method, column screening under the same conditions has been performed to determine the optimal column [16,17,18,19]. The recent development of automatic column switching has reduced the work required to screen columns. However, although this is more efficient than previous methods, it is difficult to describe this as ideal, because it is necessary to obtain a large number of columns for screening.

Classification according to selectivity simplifies the selection of stationary phases. The retention behaviors of many commercial columns used in SFC have been classified using the linear solvation energy relationships (LSER) methodology, and it is recommended to screen columns with differing selectivity [20,21,22,23,24,25,26,27,28,29]. This method is useful, but little consideration has been given to the retention factor of the stationary phase. In practical use, both selectivity and retention factor are important. Especially for SFC, CO_2_ as a mobile phase cannot be used as a sample solvent. Therefore, mismatches between the mobile phase composition and sample solvent are unavoidable. This mismatch can cause peak shape disorder and column clogging due to precipitation [30,31,32]. When an appropriate amount of modifier is added to reduce the mismatch effects, the retention factor is reduced, and thus, it is preferable for the stationary phase to exhibit a large retention factor. In addition, for online supercritical fluid extraction - supercritical fluid chromatography (SFE-SFC), which has been increasingly used in recent years [33], the retention factor of the stationary phase is important for trapping the sample.

The main reason for the difficulty in selecting a stationary phase is the ambiguous retention behavior of SFC. The above-mentioned LSER methodology has aided the elucidation of the separation mechanism of SFC, but remains incomplete [34,35]. In particular, stationary phases for both RP-HPLC and NP-HPLC, which exhibit contradictory separation properties, can be used in SFC, which is quite confusing to users familiar with HPLC [36].

Many studies have detailed the retention behaviors of stationary phases and the practicability of SFC in comparison with HPLC, where the separation mechanism is well understood. These studies mainly compared RP-HPLC using non-polar stationary phases and SFC using polar stationary phases and reported that SFC and RP-HPLC exhibit orthogonal separation and effective complementary use [8,37,38,39,40]. However, although these practicalities can be evaluated, because the mobile phase conditions of both methods differ significantly, it remains difficult to simply compare the separation mechanism without considering the mobile phase characteristics.

In SFC, CO_2_ is mainly used as the mobile phase and as CO_2_ is similar in polarity to hexane, SFC can be classified as a variant of NP-HPLC. Supercritical CO_2_ is miscible with polar solvents including methanol as well as non-polar solvents such as hexane. In NP-HPLC, a combination of a polar stationary phase and non-polar mobile phase is used, whereas in RP-HPLC, the opposite combination of a non-polar stationary phase and a polar mobile phase is employed. However, the same mobile phase can be used in SFC even with stationary phases of different polarities [41]. These features suggest that SFC features another interaction different from that of RP-HPLC or NP-HPLC. In RP-HPLC, retention is mainly caused by hydrophobic interactions, where non-polar compounds are retained longer. In contrast, in NP-HPLC, retention is mainly caused by adsorption, involving hydrophilic interactions and ion exchange, where polar compounds exhibit longer retention times. Besides these main interactions, retention can be caused by π-π and dispersion interactions. Herein, we collectively defined these non-major interactions as secondary interactions.

The π-π interactions occur between aromatic rings of organic molecules, stabilizing in a configuration where two aromatic rings overlap with a disk. Various studies have shown [42,43,44,45,46] that interactions between the π electron-rich stationary phase and solute operate in an aromatic stationary phase, such as the phenyl phase. On the other hand, dispersion interaction is an intermolecular force generated by the attraction between temporary dipoles in molecules and atoms. It has been reported [47] that this interaction is relatively strong between stationary phases containing heavy atoms or aromatic rings and the solute. We predicted that these non-major secondary interactions are key to using a non-polar stationary phase in SFC.

Herein, to simply compare the interactions occurring between the stationary phase and the solute in SFC and HPLC under similar conditions, SFC was compared with NP-HPLC using a similar polar mobile phase. Subsequently, we decided to investigate the similarities and differences between the retention behaviors of stationary phases in SFC and HPLC.

Furthermore, we clarified the retention behavior in SFC by evaluating the separation selectivity of a number of stationary phases. We aim to understand the retention factor and selectivity of each stationary phase, and to develop selection guidelines for stationary phases that are suitable for various target analytes.

## 2. Results and Discussion

### 2.1. Comparison between NP-HPLC and SFC

**Figure 1:**
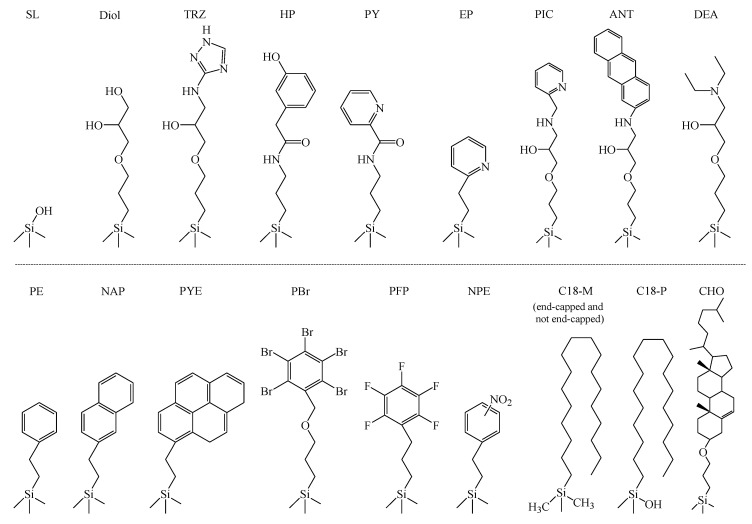
Chemical structures of the stationary phases studied herein.

**Figure 2:**
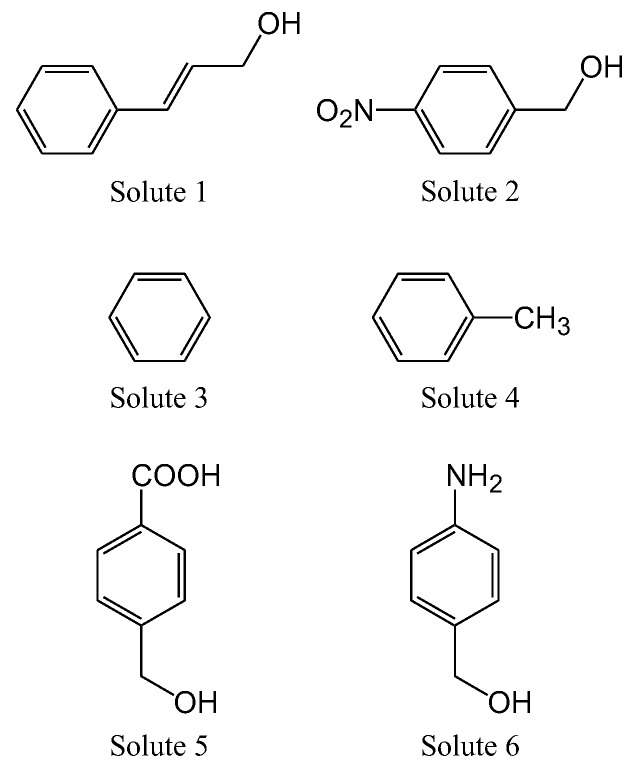
Structures of the solutes studied herein in Table 1, Table 2 and Table 3. Solute 1: cinnamyl alcohol, Solute 2: 4-nitrobenzyl alcohol, Solute 3: benzene, Solute 4: toluene, Solute 5: 4-hydroxymethylbenzoic acid, Solute 6: 4-aminobenzyl alcohol.

Figure 1 shows the stationary phase structures for all packing materials used in the present study. Various stationary phases with different properties were selected for RP-HPLC, NP-HPLC, hydrophilic interaction chromatography (HILIC), and SFC. Furthermore, in HPLC, stationary phases known to exhibit π-π and dispersion interactions were selected [42,47]. They were prepared using the same silica gel to eliminate the influence of factors other than the stationary phase, including surface properties, pore size, and surface area. As a mobile phase, we used CO_2_ for SFC and hexane with polarity close to that of CO_2_ for NP-HPLC, with ethanol as a modifier, which could mix with both the mobile phases. Figure 2 shows the test solutes used in Table 1, Table 2 and Table 3. A previous report [48] summarized the solutes used for the evaluation of HPLC, and based on the reported molecules, we selected unique evaluation solutes suitable for SFC. Solutes 1 and 2 were selected as representative polar compounds with hydroxy groups, solutes 3 and 4 as representative non-polar compounds, solute 5 as a representative acidic compound, and solute 6 as a representative basic compound. As shown in Table 1, Table 2 and Table 3, retention factors (*k*) were compared under NP-HPLC (ethanol/n-hexane) and SFC (ethanol/CO_2_) conditions for polar compounds with hydroxy groups, non-polar compounds, and ionic compounds. The mobile phases differed, but the hexane and CO_2_ concentrations were the same. For each solute, the comparison of retention factor (*k* of SFC/*k* of HPLC) is also provided. If these values are close to 1.00, they indicate similar retention behavior between SFC and HPLC. However, if the HPLC value was too small, the value is not given, as the value could not be accurately calculated (Table 2).

Polar stationary phases, including unmodified silica gel (SL), hydroxyphenyl (HP) phase, and pyridinyl (PY) phase, showed similar retention behavior in NP-HPLC and SFC (Table 1, Table 2 and Table 3). These phases retained polar solutes but not non-polar solutes. Stationary phases with acidic groups (Table 3, upper half) retained the basic compound (solute 6) to a significant extent, similar to the stationary phases with basic groups (Table 3, lower half) for the acidic compound (solute 5). These polar stationary phases retained compounds in a similar manner to those in NP-HPLC, primarily by adsorption involving hydrophilic and ionic interactions.

On the other hand, low-polarity stationary phases such as C_18_ and cholesteryl (CHO) did not retain either polar or non-polar compounds in NP-HPLC but showed slight retention in SFC (Table 2). At first glance, this seems to indicate that hydrophobic interactions contribute to retention in SFC, as in RP-HPLC. However, as the retention mechanisms of NP- and RP-HPLC are opposite, it is unlikely that they co-exist, suggesting that a different interaction dominates. This behavior is unaffected by the presence or absence of end-capping (Table 2), so it is likely that the effect originates from the C_18_ stationary phase and not that of the residual silanol.

The pyrenylethyl (PYE) and pentabromophenyl (PBr) phases, which in HPLC showed strong π-π and dispersion interactions, respectively [42,47], were found to retain solutes several times longer in SFC, indicating that these forces are enhanced in SFC compared to those in HPLC (Table 1). This is presumably because the mobile phase of SFC exhibits a lower molecular density than the HPLC mobile phase [49]. The π-π and dispersion interactions operate based on the principle of partition chromatography and are separated by differences in the distribution coefficient of the solute to the stationary and mobile phases. In SFC, since the density of the mobile phase is lower than that used in HPLC, the distribution coefficient for the mobile phase is reduced and interactions with the stationary phase are enhanced. On the other hand, adsorption chromatography, which is the major retention mechanism of NP-HPLC, separates solutes by the differences in adsorption power between the stationary phase and solute. Although the difference in adsorption due to solvation between the solvent and solute is apparent, it is presumed that large retention differences do not be observed between SFC and HPLC due to the characteristics of the stationary phase and solute.

**Figure 3:**
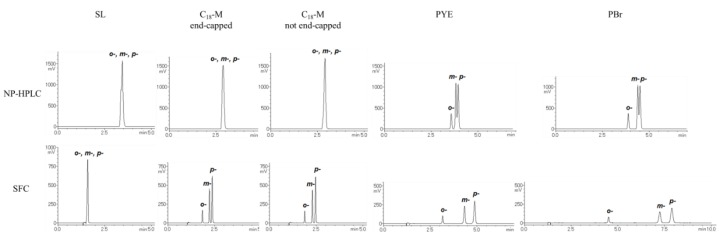
Comparison of chromatograms between normal-phase high-performance liquid chromatography (NP-HPLC) and supercritical fluid chromatography (SFC) for each stationary phase tested. Solutes: *o-*terphenyl, *m-*terphenyl, *p-*terphenyl. Upper: chromatogram of NP-HPLC. Mobile phase: ethanol/n-hexane = 10/90. Lower: chromatogram of SFC. Mobile phase: ethanol/CO_2_ = 10/90.

An excellent example showing the difference between NP-HPLC and SFC can be observed in the chromatograms of terphenyl isomers (*o-*, *m-*, *p-*), which are π electron-rich and non-polar compounds (Figure 3). In NP-HPLC, SL and C_18_ showed little retention of these compounds, whereas PYE and PBr retained the solutes slightly but with insufficient separation. In contrast, SFC with SL yielded the same result, but C_18_ retained the solute, achieving separation. In addition, the retention factors of PYE and PBr were increased and the solutes were sufficiently separated. These results are in agreement with the behavior shown in Table 1 and Table 2 and support the hypothesis that secondary interactions are enhanced in SFC compared to those in HPLC.

### 2.2. Selectivity of the Stationary Phases

**Figure 4:**
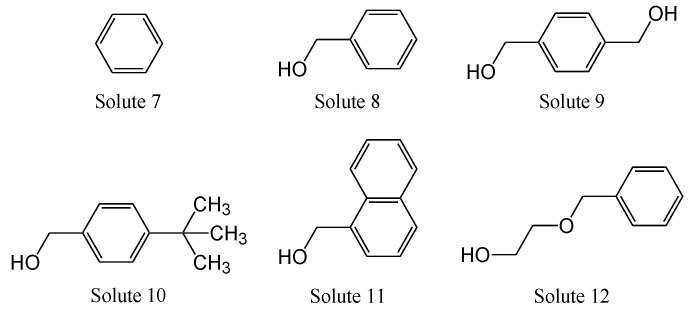
Structures of the solutes studied in Table 4. Solute 7: benzene. solute 8: benzyl alcohol, solute 9: 1,4-benzenedimethanol, solute 10: 4-tert-butylbenzyl alcohol, solute 11: 1-naphthalenemethanol, solute 12: 2-(benzyloxy)ethanol.

By evaluating the selectivity of various stationary phases in SFC, we attempted to elucidate the relevant chromatographic properties. The structures of the solutes used are shown in Figure 4. As in Figure 2, the structures were drawn to summarize the properties of the solutes used for the evaluation of HPLC. Based on these properties, we selected samples suitable for SFC evaluation. For example, benzyl alcohol with hydroxyl groups was used as the reference substance to obtain appropriate retention factors. The chromatographic properties (hydrophobic, hydrophilic, π-π, and dispersion interactions) for 18 stationary phases (Figure 1) were listed in Table 4.

Hydrophilic interactions were evaluated using the separation factor, k _Solute 9_/k _Solute 8_ (α CH_2_OH) due to the difference in terms of the hydroxymethyl group. The polar stationary phases, including Diol, HP, picolylamine (PIC), and diethylamine (DEA) phases showed high values, whereas the non-polar stationary phases, such as the C_18_ and CHO phases, exhibited low values.

**Figure 5:**
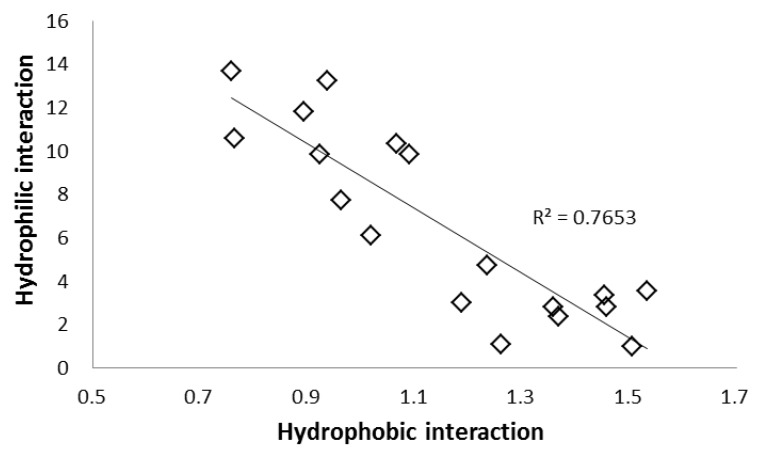
Relationship between the hydrophobic and hydrophilic interactions in SFC. Hydrophobic interaction: separation factor (α) of solutes 10/8. Hydrophilic interaction: α of solutes 9/8.

Hydrophobic interactions were also evaluated using another separation factor, *k*_Solute 10_/*k*_Solute 8_ (α tert-butyl), due to the difference in the analytes in terms of the tert-butyl group. The C_18_ and PYE phases showed high values, whereas polar stationary phases exhibited lower values. Figure 5 shows the relationship between hydrophobic and hydrophilic interactions, revealing a negative linear relationship as a matter of course. These data, in addition to the higher retention factor for solute 8 compared to that of solute 7, suggest that SFC shares a common retention mechanism as in NP-HPLC. However, this cannot fully explain the retention observed with the low-polarity stationary phases.

Next, the π-π interaction was evaluated using the following separation factor, *k*_Solute 11_/*k*_Solute 8_ (α C_6_H_4_) due to the difference of the π electron rich phenyl group of solutes 11 and 8. The PYE and PBr phases showed large values and the hydrophobic interaction was plotted against the π-π interaction, but no significant relationship was observed.

**Figure 6:**
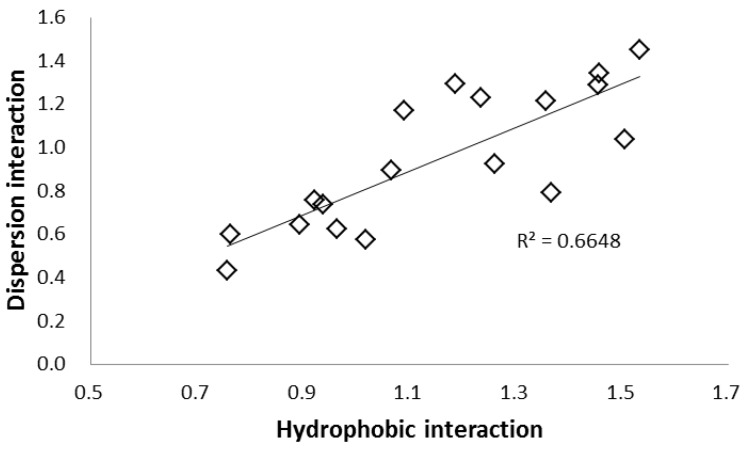
Relationship between the hydrophobic and dispersion interactions in SFC. Hydrophobic interaction: α of solutes 10/8. Dispersion interaction: α of solutes 12/8.

The dispersion interaction was evaluated using a different separation factor *k*_Solute 12_/*k*_Solute 8_ (α –OCH_2_CH-). Dispersion force generally increases with molecular size and was evaluated using solutes of similar polarity but different sizes (solutes 12 and 8). Stationary phases with polycyclic aromatic rings and heavy atoms, such as PYE, PBr and naphthylethyl (NAP) phases showed large values. On the other hand, stationary phases with monocyclic aromatic rings, such as HP, PY, and EP phases showed lower values and did not contribute significantly to dispersion interactions [46,50]. In Figure 6, the hydrophobic interaction was plotted against the dispersion interaction, and a positive correlation was obtained.

As previously mentioned, in addition to the similar retention properties of SFC and NP-HPLC, SFC-specific interactions were observed for the non-polar phases, especially PBr and PYE (Table 1 and Table 2). For HPLC, it has been reported that the PBr and PYE phases exhibit strong dispersion interactions [47]. Hydrophobic interactions, the main retention mechanism of RP-HPLC, cannot coexist with hydrophilic interactions (Figure 5). However, there is no contradiction if it is considered that the dispersion interaction, which is a secondary interaction having a positive correlation with the hydrophobic interaction, behaves like the hydrophobic interaction. These results suggest that the strong dispersion interaction is the reason that non-polar stationary phases exhibit retention in SFC.

**Figure 7:**
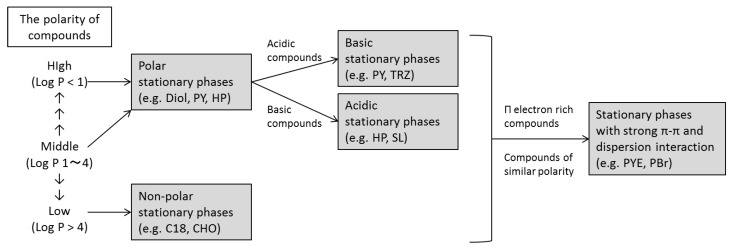
Guidelines for column selection in SFC.

From the above results, it is possible to predict a suitable column for a particular solute from the stationary phase structure. The suggested guidelines for column selection developed from the results of this study are shown in Figure 7. In creating the guideline, retention factor was more important than selectivity because, even when exhibiting high selectivity, similar compounds cannot be separated if retention is poor. If a compound has medium to high polarity (log P ˂ 4), a polar stationary phase would yield high retention, whereas compounds with low polarity (log P ˃ 4) would be better retained by a non-polar stationary phase. In contrast, if retention is excessively long resulting in elution complications, a stationary phase with a contrasting polarity could be used to decrease the retention time. In addition, by utilizing π-π and dispersion interactions, it is possible to achieve good separation in SFC for compounds that cannot be separated via HPLC.

**Figure 8:**
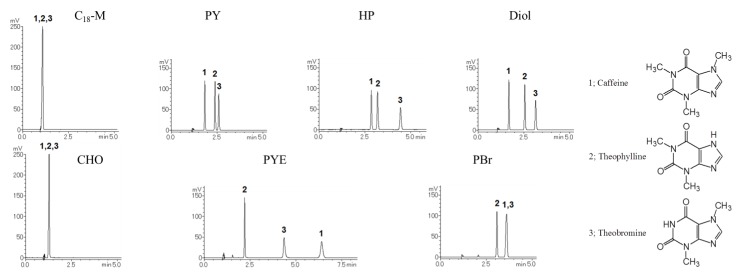
Separation of three xanthine derivatives in SFC. Mobile phase: methanol/CO_2_ = 10/90 (PYE only: methanol/CO_2_ = 20/80). Solutes: 1, caffeine; 2, theophylline; 3, theobromine.

As an indication of the differences in separation characteristics of each stationary phase, chromatograms of basic, highly polar (log P: −0.8–0.0) [51], and π electron-rich xanthine derivatives are shown in Figure 8. Although they could not be separated using low-polarity C_18_ and CHO phases, they were successfully separated using the polar HP and PY phases. In particular, they showed larger retention with an acidic HP phase. In addition, the PYE phase, which exhibits strong π-π and dispersion interactions, showed totally different separation characteristics from those of the other stationary phases. Kawachi et al. studied the retention of theobromine and theophylline using the HILIC mode with 14 different columns [52]. Comparing their results with those given in Figure 8, we noticed that SFC exhibited similar characteristics to the HILIC mode with some important differences. In the HILIC mode, in addition to the dominant hydrophilic interactions, retention was achieved by ion-exchange interactions. Theobromine (pKa = 10) is a stronger base than theophylline (pKa = 8.6), and it was reported that retention was increased in stationary phases containing acidic functional groups. In the chromatogram of SFC, shown in Figure 8, the polar stationary phases (PY, HP, and Diol) showed significant retention, but the non-polar stationary phases (C_18_ and CHO) showed poor retention. Furthermore, for the strong base theobromine, the acidic HP phase showed larger retention than that of the basic PY phase and neutral Diol phase. These retention behaviors were the same in the HILIC mode. In contrast, PYE and PBr showed large retentions despite the non-polar stationary phase, differing from the HILIC mode. These retention behaviors are due to the strong π-π and dispersion interactions in PYE and PBr, as C_18_ poorly retained theobromine and theophylline.

However, a few exceptions to these general trends were observed. For example, the aminoanthracene (ANT) phase, which, by its structure, would be predicted to exhibit strong π-π and dispersion interactions, was classified as a polar stationary phase based on its experimentally determined values, listed in Table 4. The ANT phase used herein was manufactured in two steps. The spacer was bonded to silica gel in the first step (first step phase), and aminoanthracene was bonded to the spacer in the second step (ANT phase) (Figure 1). In the evaluation in Table 4, the first step phase exhibited strong hydrophilic interactions similar to that of the ANT phase (first step phase: α = 8.95, ANT phase: α = 9.81, reference PYE phase, α = 3.54). The hydrophilic interactions in the ANT phase were slightly increased by the second reaction, but it is considered to be mainly caused by the first reaction. From the stationary phase structure, the ANT phase could be predicted to exhibit significant π-π interactions derived from anthracene. However, as shown in Table 4, the π-π interactions of the ANT phase were considerably weaker than those of the PYE phase, which contains the same polycyclic aromatic ring. Thus, it can be inferred that the unique properties of the ANT phase are a result of the small extent of anthracene binding in the second step. It is apparent that, in addition to the stationary phase structure, it is important to consider the properties and extent of the bonded phase.

## 3. Materials and Methods

### 3.1. Chemicals and Reagents

CO_2_ (99.9%), used as the SFC mobile phase, was purchased from Iwatani Corporation (Osaka, Japan). LC grade ethanol, methanol, n-hexane, and formic acid were purchased from Nacalai Tesque (Kyoto, Japan). As test solutes, cinnamyl alcohol, benzene, toluene, p-xylene, benzyl alcohol, p-terphenyl, caffeine, and theophylline were purchased from Nacalai Tesque (Kyoto, Japan), and 4-nitrobenzyl alcohol, 4-hydroxymethylbenzoic acid, 4-aminobenzyl alcohol, 1,4-benzenedimethane, 4-tert-butylbenzyl alcohol, 1-naphthalenemethanol, 2-(benzyloxy) ethanol, o-terphenyl, m-terphenyl, 1,3,5-*tri*-tert-butylbenzene, and theobromine were purchased from Tokyo Chemical Industry Co. (Tokyo, Japan). Each solute was diluted with methanol to 0.1–20 g/L to yield similar peak areas.

### 3.2. SFC Conditions

A Nexera UC SFC system, manufactured by Shimadzu (Kyoto, Japan) and equipped with a UV detector, binary pump, CO_2_ pump, auto-injector, column oven, and back pressure regulator (BPR) was used. These systems were operated with Labsolutions software provided by Shimadzu (Kyoto, Japan). All chromatographic data were collected at 40 °C with a detection wavelength of 254 nm and flowrate of 3.0 mL/min. The back-pressure regulator was set at 10 MPa, and the mobile phase compositions are listed in each table or figure. The injection volume was 5 μL, except 1 μL was used for the results listed in Table 1. The following stationary phases were obtained from commercial sources: SL, Diol, triazolyl (TRZ), HP, PY, phenylethyl (PE), NAP, PYE, PBr, pentafluorophenyl (PFP), nitrophenylethyl (NPE), monomeric C_18_ (C_18_-M), polymeric C_18_ (C_18_-P), and CHO from Nacalai Tesque (Kyoto, Japan). In addition, the following stationary phases were used as prototypes: monomeric C_18_ not end-capped (C_18_-M not end-capped), EP, PIC, ANT, and DEA from Nacalai Tesque (Kyoto, Japan). All stationary phases were prepared from full-porous silica particles (particle diameter: 5 μm, pore size: 12 nm). The non-polar stationary phases, PE, NAP, PYE, PBr, PFP, NPE, C_18_-M, C_18_-P, and CHO, were end-capped except for the specifically C_18_-M not end-capped phase. All the columns have a 4.6 mm inner diameter and length of 250 mm. During data collection, the pressures of the pumps for CO_2_ and modifier, BPR pressure, and temperature of the column were monitored to confirm stability.

### 3.3. HPLC Conditions

A Prominence HPLC system, manufactured by Shimadzu (Kyoto, Japan) and equipped with a UV detector, binary pump, auto-injector, and column oven was used. These systems were operated using Labsolutions software provided by Shimadzu (Kyoto, Japan). The flow rate was set to 1.0 mL/min and the mobile phase compositions are listed in each table or figure. The columns, detection, injection volume, and temperature conditions were identical to those used for the SFC method. During data collection, the pressure of the pump and temperature of the column were monitored to confirm stability.

### 3.4. Data Analysis

Data collection and processing were performed using Labsolutions software provided by Shimadzu (Kyoto, Japan). The retention factors (*k*) were calculated using the Equation (1) from the retention time (t_r_) and holdup time (t_0_).

*k* = (t_r_ - t_0_)/t_0_ (1)

The holdup times (t_0_) for the non-polar stationary phases including PE, NAP, PYE, PBr, PFP, NPE, C_18_-M, C_18_-P, and CHO phases were measured using first negative peak arising from the unretained dilution solvent (methanol). Because the polar stationary phases SL, Diol, TRZ, HP, PY, EP, PIC, ANT, and DEA retained methanol, t_0_ values were measured using hydrophobic compounds used in NP-HPLC such as benzene, p-xylene, and 1,3,5-tri-tert-butylbenzene.

The separation factors (α) were calculated using Equation (2) from the two retention factors.

α = *k*_1_/*k*_2_ (2)

Microsoft^®^ Excel 2010 (Microsoft Corporation, Redmond, Washington, USA) was used to construct scatter plots and calculate correlation coefficients (R^2^).

## 4. Conclusions

Here, NP-HPLC and SFC were simply compared. SFC separation conditions using polar stationary phases, such as SL and Diol, involved mainly adsorptions via hydrophilic and ionic interactions similar to those dominant in NP-HPLC. In addition to this conventional finding, non-polar stationary phases, such as PBr, PYE, and C_18,_ could be used despite the non-polar mobile phase conditions because dispersion interaction was stronger in SFC than in HPLC.

Furthermore, based on the findings presented herein, simple guidelines were created for column selection. For the LSER methodology, selection of several different cluster columns from the column classification and subsequent column screening is recommended to find the best cluster. This method requires considerable amounts of time and effort to determine the optimal column conditions for a specific analyte. In contrast, our guidelines are very simple. It is well-known that polar stationary phases are recommended for the analyses of polar compounds and non-polar stationary phases for non-polar compounds in order to increase retention. In addition, when separating compounds with aromatic rings, unsaturated bonds, or compounds of similar polarity, stationary phases with strong dispersion interaction is recommended. By analyzing various compounds according to the developed guidelines and creating a database, we believe that the practicality of SFC can be further enhanced.

Stationary phases with strong dispersion interaction retained compounds without significant contributions from polarity and may be used in a wide range of applications. This suggests that in SFC a standard column such as the C_18_ column for RP-HPLC may appear in the future.

## Figures and Tables

**Table 1:** Comparison of the retention of representative polar compounds.

Column Name	Stationary Phase (Abbreviation)	*k* (HPLC)		*k* (SFC)		*k* of SFC/*k* of HPLC	
		Solute 1	Solute 2	Solute 1	Solute 2	Solute 1	Solute 2
COSMOSIL 5SL-II	Silica gel (SL)	0.65	1.34	0.77	1.51	1.18	1.13
COSMOSIL 5HILIC	Triazole group (TRZ)	1.14	2.69	1.30	2.32	1.15	0.86
COSMOSIL 5HP	Hydroxyphenyl group (HP)	0.86	1.67	1.10	1.63	1.29	0.97
COSMOSIL 5PY	Pyridinyl group (PY)	0.82	2.26	0.92	2.07	1.12	0.91
Prototype EP	Pyridinylethyl group (EP)	0.65	1.62	0.73	1.47	1.12	0.90
COSMOSIL 5C_18_-MS-II	Octadecyl group (C_18_-M end-capped)	0.00	0.00	0.20	0.13	--	--
Prototype C_18_(not end-capped)	Octadecyl group (C_18_-M not end-capped)	0.00	0.00	0.32	0.23	--	--
COSMOSIL 5Cholester	Cholesteryl group (CHO)	0.00	0.08	0.44	0.50	--	--
COSMOSIL 5πNAP	Naphthylethyl group (NAP)	0.08	0.21	0.47	0.68	6.06	3.17
COSMOSIL 5PYE	Pyrenylethyl group (PYE)	0.13	0.51	0.81	1.66	6.40	3.27
COSMOSIL 5PBr	Pentabromophenyl group (PBr)	0.09	0.26	1.11	1.47	12.13	5.64

Mobile phase, HPLC: ethanol/n-hexane = 10/90, SFC: ethanol/CO_2_ = 10/90, *k*. Retention factors of each solute.

**Table 2:** Comparison of the retention of representative non-polar compounds.

Column Name	Stationary Phase (Abbreviation)	*k* (HPLC)		*k* (SFC)	
		Solute 3	Solute 4	Solute 3	Solute 4
COSMOSIL 5SL-II	Silica gel (SL)	0.02	0.00	0.00	0.01
COSMOSIL 5HILIC	Triazole group (TRZ)	0.03	0.01	0.00	0.01
COSMOSIL 5HP	Hydroxyphenyl group (HP)	0.02	0.01	0.00	0.02
COSMOSIL 5PY	Pyridinyl group (PY)	0.02	0.01	0.00	0.02
Prototype EP	Pyridinylethyl group (EP)	0.02	0.01	0.00	0.02
COSMOSIL 5C_18_-MS-II	Octadecyl group (C_18_-M end-capped)	0.02	0.01	0.17	0.22
Prototype C_18_ (not end-capped)	Octadecyl group (C_18_-M not end-capped)	0.04	0.02	0.07	0.12
COSMOSIL 5Cholester	Cholesteryl group (CHO)	0.02	0.01	0.11	0.17
COSMOSIL 5πNAP	Naphthylethyl group (NAP)	0.00	0.00	0.03	0.07
COSMOSIL 5PYE	Pyrenylethyl group (PYE)	0.01	0.00	0.03	0.07
COSMOSIL 5PBr	Pentabromophenyl group (PBr)	0.01	0.01	0.12	0.23

Mobile phase, HPLC: ethanol/n-hexane = 1/99, SFC: ethanol/CO_2_ = 1/99, *k*. Retention factors of each solute.

**Table 3:** Comparison of the retention of representative ionic compounds.

Column Name	Stationary Phase (Abbreviation)	*k* (HPLC)		*k* (SFC)		*k* of SFC/*k* of HPLC	
		Solute 5	Solute 6	Solute 5	Solute 6	Solute 5	Solute 6
COSMOSIL 5SL-II	Silica gel (SL)	0.66	1.24	0.81	1.32	1.23	1.06
COSMOSIL 5HP	Hydroxyphenyl group (HP)	2.19	3.31	2.24	3.27	1.02	0.99
COSMOSIL 5PY	Pyridinyl group (PY)	3.06	2.28	2.83	1.50	0.93	0.66
COSMOSIL 5HILIC	Triazole group (TRZ)	8.46	5.09	6.06	4.98	0.72	0.98

Mobile phase, HPLC: 0.5% formic acid-ethanol/n-hexane = 15/85, SFC: 0.5% formic acid-ethanol/CO_2_ = 15/85, *k*. Retention factors of each solute.

**Table 4:** Selectivity evaluation of representative compounds in SFC.

Column Name	Stationary Phase (Abbreviation)	*k* (SFC)						(1)	(2)	(3)	(4)
		Solute 7	Solute 8	Solute 9	Solute 10	Solute 11	Solute 12	Hydrophilic Interaction	Hydrophobic Interaction	π-π Interaction	Dispersion Interaction
COSMOSIL 5SL-II	Silica gel (SL)	0.00	1.06	10.42	1.16	1.85	1.24	9.83	1.09	1.74	1.17
COSMOSIL 5Diol-120-II	Diol Group (Diol)	0.00	1.50	15.82	1.14	4.46	0.90	10.56	0.76	2.98	0.60
COSMOSIL 5HILIC	Triazolyl group (TRZ)	0.00	1.76	23.23	1.65	4.29	1.29	13.22	0.94	2.44	0.74
COSMOSIL 5HP	Hydroxyphenyl group (HP)	0.00	1.31	13.52	1.40	2.98	1.17	10.33	1.07	2.28	0.90
COSMOSIL 5PY	Pyridinyl group (PY)	0.00	1.10	8.46	1.06	2.83	0.69	7.70	0.96	2.58	0.63
Prototype EP	Pyridinylethyl group (EP)	0.00	0.91	5.51	0.92	2.08	0.52	6.08	1.02	2.30	0.57
Prototype PIC	Picolylamine (PIC)	0.00	1.80	21.30	1.61	5.40	1.16	11.82	0.89	3.00	0.65
Prototype ANT	Aminoanthracene (ANT)	0.00	1.24	12.15	1.14	3.19	0.94	9.81	0.92	2.57	0.76
Prototype DEA	Diethylamine (DEA)	0.00	2.06	28.10	1.56	5.93	0.90	13.65	0.76	2.88	0.44
COSMOSIL 5PE-MS	Phenylethyl group (PE)	0.02	0.19	0.54	0.26	0.48	0.23	2.81	1.36	2.51	1.21
COSMOSIL 5πNAP	Naphthylethyl group (NAP)	0.00	0.31	0.85	0.45	0.95	0.41	2.79	1.46	3.10	1.34
COSMOSIL 5PYE	Pyrenylethyl group (PYE)	0.00	0.52	1.85	0.80	1.91	0.76	3.54	1.53	3.66	1.45
COSMOSIL 5PBr	Pentabromophenyl group (PBr)	0.04	0.71	2.37	1.03	3.67	0.91	3.34	1.45	5.19	1.29
COSMOSIL 5PFP	Pentafluorophenyl group (PFP)	0.04	0.15	0.46	0.18	0.42	0.20	3.01	1.19	2.73	1.29
COSMOSIL 5NPE	Nitrophenylethyl group (NPE)	0.00	0.50	2.36	0.62	1.27	0.62	4.70	1.23	2.54	1.23
COSMOSIL 5C_18_-MS-II	Monomeric octadecyl group (C_18_-M)	0.17	0.17	0.16	0.25	0.46	0.17	0.98	1.51	2.76	1.04
COSMOSIL 5C_18_-AR-II	Polymeric octadecyl group (C_18_-P)	0.16	0.23	0.25	0.29	0.66	0.21	1.09	1.26	2.87	0.93
COSMOSIL 5Cholester	Cholesteryl group (CHO)	0.14	0.39	0.91	0.53	1.43	0.31	2.36	1.37	3.71	0.79

Mobile phase: ethanol/CO_2_ = 5/95, (1) Hydrophilic interaction: separation factor (α) of solutes 9/8, (2) Hydrophobic interaction: α of solutes 10/8, (3) π-π interaction: α of solutes 11/8, (4) Dispersion interaction: α of solutes 12/8.
